# Mechanisms of Hepatitis C Viral Resistance to Direct Acting Antivirals

**DOI:** 10.3390/v7122968

**Published:** 2015-12-18

**Authors:** Asma Ahmed, Daniel J. Felmlee

**Affiliations:** 1 Plymouth University, Peninsula School of Medicine and Dentistry, Plymouth PL6 8BU, UK; asmaahmed1@nhs.net; 2 Plymouth University, Peninsula School of Medicine and Dentistry, Plymouth PL6 8BU, UK

**Keywords:** resistance, hepatitis C, direct acting antivirals, breakthrough variants

## Abstract

There has been a remarkable transformation in the treatment of chronic hepatitis C in recent years with the development of direct acting antiviral agents targeting virus encoded proteins important for viral replication including NS3/4A, NS5A and NS5B. These agents have shown high sustained viral response (SVR) rates of more than 90% in phase 2 and phase 3 clinical trials; however, this is slightly lower in real-life cohorts. Hepatitis C virus resistant variants are seen in most patients who do not achieve SVR due to selection and outgrowth of resistant hepatitis C virus variants within a given host. These resistance associated mutations depend on the class of direct-acting antiviral drugs used and also vary between hepatitis C virus genotypes and subtypes. The understanding of these mutations has a clear clinical implication in terms of choice and combination of drugs used. In this review, we describe mechanism of action of currently available drugs and summarize clinically relevant resistance data.

## 1. Introduction

Hepatitis C virus (HCV) infection is a major global health problem and a leading cause of morbidity and mortality. The most recent estimate showed an increase in the prevalence of HCV infection over last 15 years from 2.3% to 2.8%. This equates to 170 million people who are chronically infected worldwide and 3–4 million developing new infection with HCV each year [1,2] while 350,000 people die every year due to HCV related complications [3].

Following exposure to HCV, only a minority of cases are able to clear the virus spontaneously. The majority of individuals (approximately 80%) develop chronic infection with persistent viremia and chronic hepatitis. This frequently results in the development of progressive liver fibrosis and ultimately cirrhosis, with its attendant risks of developing liver failure and hepatocellular cancer [2,4].

Until 2011, HCV standard-of-care treatment consisted of interferon alpha and ribavirin for several months, which is associated with detrimental side effects affecting compliance and poor outcomes. However, new and promising direct-acting antiviral agents (DAAs) have recently become available with more in the development pipeline resulting in a remarkable transformation in treatment of HCV. DAAs are drugs targeting specific HCV encoded proteins resulting in disruption of the viral life cycle. A number of DAAs are either approved or in phases of advanced development and clinical trials. The first generation of DAAs was administered in conjunction with pegylated interferon, so while the efficacy of treatment increased, the issues with side-effects remained. However, the incorporation of next-generation DAAs into the antiviral cocktail is leading to interferon-free regimens in clinical practice. Although the first generation of DAAs (NS3/4A inhibitors Telaprevir and Boceprevir) were co-administered with pegylated interferon and ribavirin, thereby adding to the side effect burden [5,6], the second generation of DAAs have minimal side effects, are efficacious with shortened courses of treatment, and are associated with cure rates of more than 90% in phase II and III studies.

The initial observations with these DAA regimens in various real-world cohorts also show high SVR rates of 80%–90% but they are slightly lower than those seen in registration studies. For example, the first approved interferon-free regimens for treating genotype 2 and 3 infection included a combination of sofosbuvir (an NS5B inhibitor) and ribavirin for 12 to 24 weeks. This resulted in an SVR of 68%–90% [7,8,9,10]. DAA combinations currently recommended to treat genotype 3 infection include NS5B polymerase inhibitor sofosbuvir and NS5A inhibitor daclatasvir with or without ribavirin while a combination of sofosbuvir and ribavirin is recommended for treatment of HCV genotype 2 infection, with possible addition of pegylated interferon alpha in patients with previous treatment failure. Other DAA combinations available to treat genotype 1 infection include sofosbuvir with either of the two NS5A inhibitors daclatasvir or ledispavir, a regimen consisting of ombitasvir-paritaprevir-ritonavir, and either dasbuvir or simeprevir with sofosbuvir. Treatment choice depends on HCV genotype, presence of cirrhosis, Child Pugh Class and previous HCV treatment experience. The above findings and extensive use of these drugs in the near future predicts that a proportion of patients will fail to achieve SVR and develop resistance.

HCV exists as a heterogeneous pool of genetic variants within an infected individual prior to treatment. This is due to the high error rate of HCV polymerase introducing on average one mutation per replicant and high rate of virion production [11,12]. Certain polymorphisms, which are resistant to direct acting antivirals, can exist at low levels prior to treatment and may get selected upon exposure to these drugs. In this review, we discuss DAAs according to their mechanism of action, drug resistance profiles, and discuss clinical applications where relevant.

## 2. Emergence of DAA Resistance

HCV has higher sequence diversity even within an individual genotype in comparison to other chronic viral infections such as hepatitis B virus or HIV [13]. HCV has a high turnover rate with an estimated half-life of only 2–5 h with 10^10^ to 10^12^ virions produced and cleared per day in an infected patient [11,14,15,16]. Because of lack of proof reading activity of HCV RNA dependent RNA polymerase (NS5B) [12,17] and high replication activity of HCV, a large number of viral variants are produced continuously during infection with an error rate of 10^−3^ to 10^−4^ mutations per nucleotide per genomic replication [17]. Most of these variants are cleared by the host immune system or are unable to replicate because of a functional loss in encoded proteins [18,19]. Thus a heterogeneous mixture of closely related genomes comprising a dominant strain (wild type strain) along with other strains present at lower frequencies makes up the HCV population in a given host. This pool of variants is termed the quasispecies in the host. This quasispecies existence of HCV in a given host results in a significant adaptation advantage because the simultaneous presence of multiple variant genomes allows for on-going evolutionary selection of mutations with better fitness to any given condition. A classic example of this is the adaptation of the quasispecies that occurs post-liver transplantation [20]. Hence, HCV variants with a different level of susceptibility may exist naturally at low levels in the absence of drug pressure and can be selected in patients with suboptimal response to treatment [21]. The biological and clinical implication of this selection is resistance to direct acting antiviral agents and treatment failure.

Relapse of HCV in patients who initially respond to DAA may be due to the replication of a residual variant that remained below the limit of detection at the end of treatment. Viral sequencing at the time of relapse may identify the virus sequence present at the end of treatment but it is also possible for viral population to evolve to wild type prior to sequencing after relapse, as it is not under selective pressure at the time.

## 3. Direct Acting Antiviral Drugs

**Figure 1:**
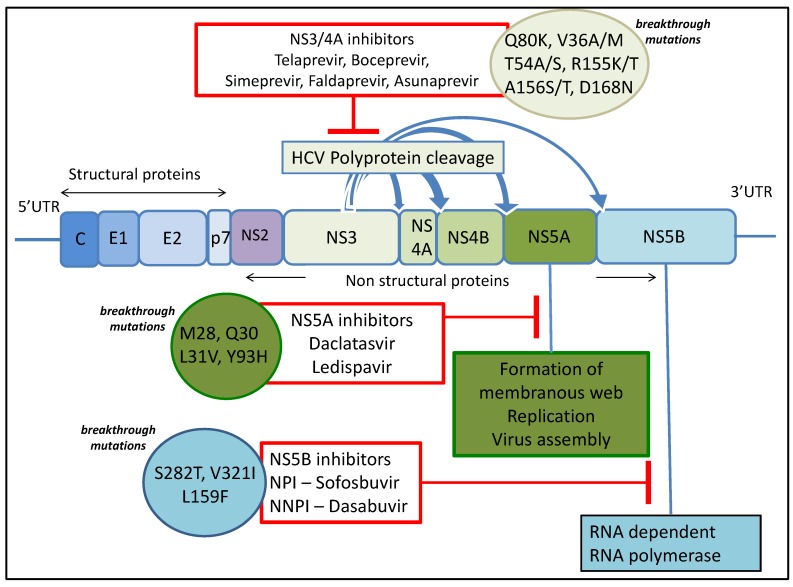
The HCV genome encodes a single open reading frame flanked by 5’ and 3’ untranslated regions (UTR). Cleavage of this polyprotein by host endopeptidases (for structural proteins) and viral non-structural protein NS3/4A results in producing structural proteins core (C), Envelope proteins 1 and 2 (E1, E2), and non-structural proteins p7, NS2, NS3, NS4A, NS4B, NS4A, and NS5B. Enzymatic function of non-structural proteins has been the basis of DAAs (outlined in red). These can be overcome by common breakthrough mutations (circled and highlighted).

The HCV genome is composed of a 9kb open reading frame flanked by a 5’ and 3’ untranslated region. The genome is translated into a polyprotein that is cleaved to form structural (Core, E1, E2) and non-structural proteins (p7, NS2, NS3, NS4a, NS4b, NS5A and NS5). DAAs have been developed to target non-structural viral proteins involved in viral replication. These include NS3-4A inhibitors, NS5A inhibitors and nucleoside and non-nucleoside inhibitors of NS5B (Figure 1). However, studies have shown that various breakthrough viral mutations in viral proteins can confer resistance to the respective DAAs (Figure 1).

The mechanism of action of these DAAs and evidence for breakthrough resistance mutations in the light of current evidence is discussed below.

### 3.1. NS3-4A Protease Inhibitors

The target for these agents is the viral protease NS3/4A essential for cleaving the HCV encoded polyprotein into individual viral proteins facilitating replication. It is a heterodimer complex of NS3 and NS4A proteins. NS3 possesses the proteolytic site while NS4 is a cofactor. The NS3/4A protease cleaves the HCV polyprotein generating non-structural viral proteins NS3, NS4A, NS4B, NS5A and NS5A. NS3/4A inhibitors block the NS3 catalytic site or inhibit NS3/4A interaction thereby blocking HCV polyprotein cleavage [22].

In addition to this direct action, they may also help recover innate immune processes. NS3/4A protease blocks Toll/interleukin-1 receptor domain-containing adaptor inducing IFN-β (TRIF) mediated Toll-like receptor signalling, as well as mitochondrial antiviral signalling protein (MAVS, also termed IPS-1, VISA, and CARDIF) mediated RIG-I signalling [23,24] which then results in impaired interferon induction [25]. Therefore, it is possible that inhibition of this protease also helps recover interferon production.

The catalytic site on NS3 protease consists of the amino acid triad serine-histidine-aspartate and is located in a shallow substrate binding groove with solvent features which does not facilitate tight binding to the inhibitors [26]. Therefore, inhibitors depend on few interactions with the enzyme and only a few critical mutations in the enzyme may be enough to confer significant resistance to these drugs [27,28,29].

First generation NS3/4A protease inhibitors include boceprevir and telaprevir which were the first DAA approved for treating chronic HCV genotype 1 and achieved significantly higher SVR rates when given in combination with pegylated interferon and ribavirin compared to previous standard treatment consisting of pegylated interferon and ribavirin alone. They are given in combination with pegylated interferon and ribavirin as the efficacy of these combination regimens still largely relies on the viral sensitivity to interferon. This combination has shown a higher SVR in previous partial responders or relapsers to combination of pegylated interferon and ribavirin than with null responders. The virus has a low genetic barrier to development of resistance to both boceprevir and telaprevir. Baseline sequencing analysis for the presence of amino acid substitutions in the NS3/4A region has shown detectable resistance associated variants (RAVs) in patients with HCV genotype 1 pre-treatment [27,28,30]. These are present in 8.6% of treatment naïve GT1a and 1.4% of treatment naïve GT1b population [30]. However, their presence did not always preclude treatment success [27,28]. On the other hand, the majority of patients who did not achieve SVR had detectable resistant variants at the time of treatment failure [27]. Baseline testing for these variants regarding clinical outcome is therefore not recommended [31,32].

Analysis of HCV sequences in patients who did not achieve SVR with telaprevir based regimen in phase 2 and 3 studies identified following variants as being significantly enriched: V36A/M, T54A/S, R155K/T, A156S/T and D168N [27]. These amino acid positions associated with resistance are located near the protease catalytic site in the NS3 protease domain, consistent with the mechanism of action of a protease inhibitor. Kieffer *et al.* [27] in the same study also performed enzymatic and replicon based phenotypic studies to show that these mutations confer different levels of resistance to telaprevir *in vitro*. Variants conferring low level resistance were defined as a 3–25 fold increase in IC_50_ from wild-type whilst those conferring high level had >25 fold increase in IC_50_. Interestingly, almost all the viral breakthroughs during telaprevir treatment were associated with high-level resistant variants [27].

Breakthrough variants tend to be less efficient at replication, therefore they are replaced by primary variants once selection pressure from treatment ceases. Sequence analysis of HCV during follow up from patients who did not achieve SVR in phase 3 studies of telaprevir based therapy suggests replacement of resistant variants with wild type virus over a median time period of 10 months for genotype 1a and three weeks for genotype 1b infection [27,33,34]. It may therefore be possible to treat these individuals with alternative treatment options.

Second-generation NS3/4A protease inhibitors have improved potency but the problem of low genetic barrier to developing resistance remains with these agents. These second generation inhibitors include simeprevir, paritaprevir and asunaprevir. Asunaprevir is available in Japan, whilst grazoprevir is in phase 3 clinical trials. Population based sequencing identified a key clinically relevant polymorphism Q80K which was found in 19%–48% of NS3 protease sequences from genotype 1a [35]. *In vitro* studies have shown that Q80K decreases viral susceptibility to simeprevir by 10-fold [36]. Less profound decreases in viral susceptibility were seen to other second line NS3 inhibitors including sovaprevir and asunaprevir [37]. These findings were supported *in vivo* in phase 2 and phase 3 clinical trials assessing simeprevir with pegylated IFN and ribavirin in genotype 1 patients [35,38]. Patients infected with HCV genotype 1 with baseline Q80K polymorphism have a significantly lower rate of achieving SVR relative to those without this polymorphism (58% *vs.* 84%). It is therefore clinically recommended to perform baseline resistance testing for this mutation in genotype 1a patients and to avoid simeprevir treatment when this polymorphism is present. A recent interim analysis from an open label study which assessed simeprevir in combination with daclatasvir and sofosbuvir in a small number of patients with advanced liver disease showed that all patients achieved SVR12 (sustained viral response at 12 weeks post treatment) including patients with baseline Q80K or NS5A polymorphism showing the strength of combinatorial treatment [39].

Resistance mutations emerging during unsuccessful treatment with first-generation protease inhibitors have been associated with decreased *in vitro* susceptibility to simeprevir and are therefore also expected to have an impact on clinical outcome.

### 3.2. NS5A Inhibitors

NS5A protein is involved in viral replication, assembly, and release of HCV particles [40,41,42]. NS5A protein has three domains. These are N terminus domain I (amino acids 1–213), domain II (amino acids 250–342) and C–terminus domain III (amino acids 356–447) [43]. Domains I and II are involved in RNA replication while domain III is essential for virion assembly. NS5A inhibitors such as daclatasvir block replication of HCV RNA as well as virus assembly. In particular, inhibitor binding to NS5A results in conformational changes thereby preventing NS5A interaction with membranous and cellular proteins. This, in turn, abrogates the formation of membranous web, which is the virus induced membrane compartment where RNA replication occurs. [41,42,44,45]

Currently, available NS5A inhibitors are daclatasvir, ledipasvir and ombitasvir. The latter two are available in fixed dose combinations with other direct acting antiviral agents. Elbasvir and Veltapasvir are being studiedin phase 3 clinical trials in combination with NS3 inhibitor grazoprevir and the NS5B inhibitor sofosbuvir, respectively. Amongst these, daclatasvir targets NS5A domain I. Although NS5A inhibitors are quite potent and have a broad genotypic coverage, they are also associated with a relatively low viral barrier to resistance and long-time persistence of RAVs.

Daclatasvir is associated with a higher response in genotype 1b compared to 1a, which is also explained by the higher barrier to resistance by genotype 1b. In genotype 1a, selection of a single mutation is enough to lose susceptibility to daclatasvir [46,47]. In genotype 1b, single amino acid substitution and some double amino acid substitutions (Q54H-Y93H) conferred minimal resistance. However, some double substitutions (L31V-Y93H) in G1b are associated with a high level of resistance [47]. Polymorphisms of NS5A protein that have been associated with resistance both *in vitro* and *in vivo* include variants at amino acids M28, A30, L31 and Y93 for genotype 1a and L31 and Y93 for genotype 1b [47].

Naturally occurring polymorphisms in NS5A may also influence susceptibility to daclatasvir. Such polymorphisms are less common in genotype 1a and 3 but much more common in genotype 1b and 4. In genotype 2, mutation L31M is seen in 50%–85% but clinical trials have shown that it does not predict treatment failure in a study where it was given in combination with pegylated interferon alpha and ribavirin [48]. Subsequent Phase III clinical trials of daclatasvir with asunaprevir (NS3/4A inhibitor) have shown that the presence of baseline polymorphisms at amino acids L31 and Y93 is associated with loss of susceptibility to NS5A inhibitors [49].

A recent large study evaluating NS5A RAVs in samples from genotype 1a infected patients from 22 different countries treated with a combination of sofosbuvir and NS5A inhibitor ledipasvir did not show any difference in baseline prevalence of these variations between different regions and ethnicities. However, in genotype 1a patients, lower SVR 12 rate was observed in patients with pretreatment NS5A resistant associated variants conferring high level (>1000 fold) resistance to NS5A inhibitors when treated for 24 weeks. These included H58D, Y93H/N/F or multiple RAV combinations. The largest impact of RAVs on treatment outcome was observed in patients with cirrhosis treated for 24 weeks with sofosbuvir and ledipasvir. However, SVR rates were similar in genotype 1b patients with and without pretreatment NS5A RAVs. The same study also reported that IL28B-CC genotype is significantly associated with a higher prevalence of Y93H [50]. This association has been reported previously but currently not functionally understood and clinical relevance needs to be defined further. This may, however, explain a lack of or even inverse correlation of treatment response with IL28B genotype in some NS5A inhibitor containing IFN-free regimens [51].

One of the key resistance determinants associated with amino acid substitution, Y93H was also detected as a natural baseline polymorphism in 13 out of 148 genotype 3 patients in a clinical trial of daclatasvir and sofosbuvir. This was also associated with lower SVR rates [52]. The relevance of breakthrough mutations in the context of NS5A inhibitors is reflected in that common mutational analysis is commercially available in the USA, although baseline testing is not currently recommended by guidelines. DAA combinations that target multiple targets and include sofosbuvir may be a strategy to treat patients with NS5A resistant associated variants.

An emerging problem that has been revealed with NS5A inhibitors is the extensive persistence of NS5A RAVs post-treatment, possibly even indefinitely [53]. However, renewed hope in NS5A inhibitors is restored with a recent report that daclatasvir when used with an analogue molecule restores its potency >1000 fold presumably through NS5A-NS5A interactions [54].

It is probable that due to protein-protein interactions during HCV replication, mutational changes in other proteins including NS3, NS4B and NS5B can also influence susceptibility to NS5A inhibitors. The analysis of single breakthrough mutations may be simplistic given that these non-structural proteins work harmoniously for efficient HCV replication. Likewise, the role of secondary structure within the RNA genome is also a consideration in replication efficiency.

### 3.3. NS5B Inihibitors

NS5B is an RNA dependent RNA polymerase which is involved in the processing of the viral RNA genome to make a negative strand template and subsequently transcription of daughter copies of the genome [55]. The enzyme is structurally organised in a characteristic “right hand motif” which contains palm and thumb domains. It has two sites: a catalytic site for nucleoside binding and four other sites responsible for allosteric alteration where non-nucleoside compounds can bind. Therefore, there are two classes of polymerase inhibitors: nucleoside analogues also called nucleoside polymerase inhibitors (NPI) and also non-nucleoside analogues also called non-nucleoside polymerase inhibitors (NNPI).

The structure of RNA dependent RNA polymerase is highly conserved across all HCV genotypes, thereby making these agents efficacious against all six HCV genotypes [22].

#### 3.3.1. Nucleoside Polymerase Inhibitors (NPI)

NPIs are activated within hepatocytes through phosphorylation to nucleoside triphosphate, which competes with nucleotide substrates that are incorporated into the nascent RNA chain resulting in chain termination during RNA replication of viral genome [56].

The advantage of these agents is a very high barrier of viral genetic resistance. The active (catalytic) site of NS5B is relatively intolerant to amino acid substitutions. Hence, active site mutations that confer resistance to NPI are also likely to impair RNA polymerase activity compared with the mutations in NNPI allosteric binding sites thereby making the mutant virus less fit compared with the wild type virus by seriously impairing viral replication. S282T is one such mutation that was idenfitied *in vitro*, however it has rarely been detected in patients with treatment failure in clinical trials as mentioned later in this article.

Sofosbuvir is the first NS5B NPI to become available. Resistance variants within NS5B polymerase have been reported with *in vitro* exposure to sofosbuvir although their clinical significance is not entirely clear. These variants include S282T, L159F and E341D. *In vitro* studies using HCV replicon systems, S282T was most commonly selected in all genotypes but mainly conferred resistance to G1a and G1b with only a modest resistance in G2a replicon harbouring this mutation [57]. These studies also showed that although S282T mutation was selected alone in G1b, in G1a an additional mutation I434M was selected along with S282T on exposure to sofosbuvir. In the case of G2a, S282T together with other mutations T179A (finger domain), M289L and I293L (palm domain) were essential for conferring resistance to sofosbuvir.

Amongst these, S282T mutation has infrequently been associated with reduced susceptibility to sofosbuvir *in vivo* [58]. In the ELECTRON sofosbuvir monotherapy study, the S282T substitution was detected in a patient infected with HCV genotype 2 who had a virological relapse Week 4 post treatment [59]. It was also detected in a single patient who relapsed after 24 weeks treatment with sofosbuvir and ribavirin who had virological relapse in the National Institutes of Health (NIH) SPARE trial [60]. In a pooled analysis of sofosbuvir phase 3 trials involving patients infected with HCV genotype 3 who did not achieve SVR, the substitutions L159F and V321A were selected post baseline in several cases. In addition, substitutions C316N/H/F were present at baseline in in six patients with HCV genotype 1b who failed treatment and one patient with genotype 1a who experienced relapse [56]. In another recent pooled analysis of phase 2 and 3 studies where sofosbuvir based regimen was administered, no S282T variant was detected at baseline. Emergence of this variant was infrequent (1%) in subjects who had virlogical failure. S282T levels declined on average by four fold within two weeks of follow up period confirming low replication fitness of this variant [61].

#### 3.3.2. Non-Nucleoside Polymerase Inhibitors (NNPI)

Non-nucleoside polymerase inhibitors interact with RNA-dependent RNA-polymerases at one of the four non catalytic allosteric binding sites and prevent conformational changes in the enzyme crucial for its function. They can be subclassified based on their allosteric binging site: Palm 1, Palm 2, Thumb1 and Thumb2 [62].

These agents have lowest barrier to resistance amongst all DAA. Consequently, they have been used and studied in combination with other DAA as a part of multitarget strategy involving non-overlapping drug targets to overcome RAVs to individual drugs. They do not exhibit cross resistance with NPI given their different site and mechanism of action. Examples of these agents are dasabuvir, which acts at Palm 1 site of RNA polymerase, and Baclabuvir, which acts at Thumb 1 site.

*In vitro* studies of dasabuvir using genotype 1 replicons showed that C316Y variant in both genotype 1a and 1b and Y448C/H in genotype 1b conferred >900 fold resistance to dasabuvir. Other amino acid substitutions at positions 368, 411, 553, 556 and 559 were associated with lower level of resistance. Out of these variants, C316, M414T, Y448H, S556G had higher replication capacity than other variants making them more likely to cause resistance in clinical practice [63].

Dasabuvir is administered in combination with ombitasvir-paritaprevir-ritonavir. This regimen is referred to as 3D regimen. Ombitasvir is an NS5A inhibitor, paritaprevir is an NS3/4A inhibitor and ritonavir is a potent inhibitor of CYP3A4 and a pharmacological booster of paritaprevir. In phase 2 and 3 clinical trials treating HCV genotype 1 infection with 3D regimen and ribavirin resulted in 95%–98% SVR [64,65]. This suggests that a multitargeted approach may maximize the response rate. However, exposure to ombitasvir, paritaprevir, and dasabuvir can select for mutations in the NS5A, NS3, and NS5B, respectively, thereby decreasing the activity of the specific agent in this combination. In clinical trials, the most common mutations that emerged during treatment or on relapse among subtype 1a infections were D168V in NS3, M28A/T/V and Q30E/K/R in NS5A, and S556G/R in NS5B [65,66,67,68]. Mutations seen in genotype 1b relapse were Y56H and D168V in NS3, L31M and Y93H in NS5A and S556G in NS5B at the time of relapse [65]. Consistent with the concept that multi-targeted approaches reduce viral breakthrough, next-generation sequence analysis of patients in the COSMOS study that included simeprevir and sofosbuvir with or without ribavirin revealed a lack of association of treatment response to well-characterized resistance mutations [69].

## 4. Prevalence before and after Treatment with DAA

Under the selective pressure of DAAs, viruses with RAVs emerge that are undetectable prior to therapy. In a landmark study, Sarrazin *et al* [70] found that treatment with telaprevir resulted in the emergence of low-level resistance (V36A/M, T54A, R155K/T, A156S) and high-level resistance (A156V/T, 36 + 155, 33 + 156) RAVs with had frequencies inversely correlating with resistance. These variants were detectable using subcloning up to seven months after the cessation of therapy, implying that the minor variant may persist much longer. Similar RAVs were verified and revealed by applying similar methods for the other first-generation protease inhibitor, boceprevir, including V55A [71]. Approximately 50% of patients that fail treatment with boceprevir have detectable RAVs [32]. Next generation sequencing (NGS) analysis of a small number of individuals that repeatedly failed telaprevir treatment surprisingly did not have persistent RAVs present, but apparently independently experienced a de novo RAV generation upon treatment [72]. However the limitation of this study was 1% frequency, and recent evidence suggests that abundancies below 0.02% may be relevant for emergence of RAVs [73].Even in high-risk populations, treatment failure is more associated with the emergence of a pre-existing minority variant rather than reinfection [73].

A very recent analysis attempted to refine some of the NGS data detected at least low levels of susceptible or moderate resistance RAVs to second-generation protease inhibitor, simeprevir, in each patient analysed [74]. The prevalence of the common NS3 Q80K RAV that affects simeprevir efficacy is dependent on subtype and ethnic prevalence [75].

The most successful NS5B inhibitor in treatment now is sofosbuvir. Prior to full clinical development, the S282T RAV appeared to be problematic. However, this variant appears very rare [76,77]. Donaldson *et al* [74] performed an analysis on four phase III clinical trials in search of common RAVs against sofosbuvir, discovering L159F, C316N, and V321A were associated with virological failure [78]. Interestingly, this study also verified S282R mutation as associating with failure. It should be noted that the majority of patients with relapse had no clear resistance variants emerge, however there is the possibility that this is due to a lack of sensitivity in NGS technique.

NS5A RAVs can be very common, with Y93H detected in up to 15% of the population and L31M in up to 6.3% [79]. Other RAVs tend to also be fairly common detected in approximately 0.3%–3.5% of the population. Substitutions in genotype 1a include M28T, Q30R/H, L31V, and Y93R. Resistant variants persisted in the population beyond six months after treatment, revealing that these variants are well tolerated [80]. This represents a major challenge as most of the next-generation formulations include an NS5A inhibitor and there are some estimations that NS5A RAVs could persist indefinitely [53]. Recent compelling evidence shows that daclatasvir treatment of an NS5A inhibitor resistant variant in combination with an analogue of daclatasvir, dramatically enhances the resistance barrier [54]. This is due to communication between NS5A molecules resulting in allosteric differences in inhibitor binding. Table 1 summarises the data for most prevalent resistance associated variants and drug class.

**Table 1:** Common RAVs; resistance and prevalence.

Drug Class	Example	Common RAV	Resistance	Prevalence
NS3/4A inhibitor	telaprevir	V36M	low	<1%
	boceprevir	T54S/A	low	2%–3%
	simeprevir	V55A	low	0.4%–3%
	asunaprevir	Q80K	low	0.5%–75%
	faldaprevir	R155K	high	<1%
NS5A inhibitor	daclatasvir	M28	high	0.5%–4%
	ombitasvir	Q30	high	0.3%–1.3% (geno 1) 50%–100% (geno 3,4)
	ledipasvir	L31V	high	0.9%–6.3% (geno 1) 74%–100%(geno 2,4)
		Y93H	high	1.5%–14%
NS5B NPI	sofosbuvir	L159F	n.d.	5.2%
		V321A	n.d.	2.2%
		S282R	low	0.4%
NS5B NNPI	dasabuvir	C316N	low	11%–36%

Data was summarised and collated for the most prevalent RAVs [78,79,81,82]. n.d.: no data.

## 5. Clinical Significance of Baseline RAVs

In sofosbuvir trials, while there were variants that emerged and were statistically significant with resistance, the majority of subjects that experienced relapse did not carry identifiable RAVs [78]. For other drug classes, the emergence of resistant variants may be derived from a very small proportion of the quasispecies that can only be detected with NGS, accompanied with costly analysis. Our current understanding of the breadth and strength of resistance, in combination with contributing host responses make response prediction based on sequence analysis untenable [83]. At this time, data need to be collected on all of these classes of drugs and the next-generation of agents in various combinations for comprehensive understanding of how to minimize or ablate breakthrough mutations.

## 6. Conclusions

HCV drug resistance is an important and upcoming clinical issue in the context of limited data and a large number of DAA in development or approved for clinical use. Understanding the position and mechanism of resistance may result in engineered antiviral cocktails that are highly efficacious and minimize side-effects. The relevance of pre-existing resistance mutations for response to DAAs needs to be better studied in order to understand their significance in selective tailoring of various DAA for personalized care.
